# The ‘Dr Jekyll and Mr Hyde fungus’: noble rot versus gray mold symptoms of *Botrytis cinerea* on grapes

**DOI:** 10.1111/eva.12079

**Published:** 2013-07-15

**Authors:** Elisabeth Fournier, Pierre Gladieux, Tatiana Giraud

**Affiliations:** 1 Biologie et Génétique des Interactions Plante-Parasite, INRA-CIRAD-SupAgroMontpellier Cedex 5, France; 2 Ecologie, Systématique et Evolution, Université Paris-SudOrsay Cedex, France; 3 Ecologie, Systématique et Evolution, CNRSOrsay Cedex, France; 4 Plant and Microbial Biology, University of CaliforniaBerkeley, CA, USA

**Keywords:** Ascomycete, *Botrytis cinerea*, genetic structure, gray mold, microsatellites, noble rot, population structure

## Abstract

Many cryptic species have recently been discovered in fungi, especially in fungal plant pathogens. Cryptic fungal species co-occurring in sympatry may occupy slightly different ecological niches, for example infecting the same crop plant but specialized on different organs or having different phenologies. Identifying cryptic species in fungal pathogens of crops and determining their ecological specialization are therefore crucial for disease management. Here, we addressed this question in the ascomycete *Botrytis cinerea*, the agent of gray mold on a wide range of plants. On grape, *B. cinerea* causes severe damage but is also responsible for noble rot used for processing sweet wines. We used microsatellite genotyping and clustering methods to elucidate whether isolates sampled on gray mold versus noble rot symptoms in three French regions belong to genetically differentiated populations. The inferred population structure matched geography rather than the type of symptom. Noble rot symptoms therefore do not seem to be caused by a specific *B. cinerea* population but instead seem to depend essentially on microclimatic conditions, which has applied consequences for the production of sweet wines.

## Introduction

Since the advent of molecular biology, the use of genetic markers within the population genetics framework has revealed the existence of many cryptic species, that is*,* species previously unrecognized based on morphology alone, in a wide range of organisms (e.g., Taylor et al. 2000; Gentetaki and Lynn 2010; Heimeier et al. 2010; Jesse et al. 2010; Kreier et al. 2010; Nygren et al. 2010; Gladieux et al. 2011a,b; Liu et al. 2011; Piggott et al. 2011). This is especially true for microorganisms like fungi, because few morphological traits can be used to discriminate species and because inducing mating *in vitro* to test for interfertility is often impossible or less discriminant than other species criteria (Koufopanou et al. 1997; Dettman et al. 2003a,b; Fournier et al. 2003; Le Gac et al. 2007a,b; Giraud et al. 2008). Some of the recently discovered fungal cryptic species have been found to occur in allopatry, for instance on different continents, such as in *Neurospora crassa* (Dettman et al. 2003a), yeasts (Kuehne et al. 2007), or the crop pathogen *Fusarium graminearum* (O'Donnell et al. 2004). Other fungal cryptic species occur in sympatry and often display seemingly similar ecological niches (Fitt et al. 2006; Le Gac et al. 2007a; Giraud et al. 2008). In this latter case, the sympatry between closely related species with apparently similar ecological niches can be explained by a recent secondary contact after speciation in allopatry or by different resources use (Giraud et al. 2008; Gladieux et al. 2011b). For instance, in fungal plant pathogens, sibling species infecting the same crop plants are often specialized on different organs or have different phenologies (Fitt et al. 2006). Beyond the fundamental interest in understanding the coexistence of sibling species in ecology, it is essential for disease management to recognize the different cryptic species in fungal pathogens of crops and their ecological specialization, as they can exhibit differences in fungicide resistance (Fournier et al. 2005), phenologies (Walker et al. 2011), organ specialization (Fitt et al. 2006), host specialization (Le Gac et al. 2007a; Fournier and Giraud 2008), or ability to infect resistant plant varieties (Le Cam et al. 2002; Gladieux et al. 2010). Recognizing sibling pathogen species with such specialization has obviously important applied consequences for their control. An efficient control program demands good knowledge of the targeted species, in terms of fungicide resistance, phenology, and potential gene flow from related species (i.e., potential introgression of genes controlling fungicide resistance, aggressiveness, or virulence). For example, rapid and efficient diagnostics of *Candida* cryptic species allowed demonstration of differences in their prevalences in immunocompromised humans and in their susceptibility profiles to antifungal compounds (Johnson 2009; Peman et al. 2012). Such discoveries help targeting treatments against the most harmful species. The recognition of cryptic species is also critical in pathogen species useful to humans. An example is the understanding of the environmental distributions of the entomopathogenic fungus *Metarhizium anisopliae* and its sister cryptic species, widely used as biological control agents for insects (Schneider et al. 2011).

Elucidating the population structure of pathogenic fungi is of uttermost importance when the targeted species or species complex can be at the same time a pest and a benefit, such as the ascomycete *Botrytis cinerea*. This ‘Jekkyl and Hyde’ fungus is indeed responsible for the gray mold disease that causes important damage on numerous host plants such as grapevine, strawberries, or tomatoes, but it is also responsible for the noble rot on grape berries used for processing sweet wines such as Sauternes. Despite its great economic importance, the difference between the noble rot and gray mold symptoms on grapevine has been little investigated. Grapevine noble rot, also called ‘botrytization’ or ‘over maturation’ of perfectly ripe berries, is a progressive process taking place at the end of the growing season, lasting 10–20 days and being restricted to particular ‘terroirs’ (i.e., particular combinations of pedo-climatic conditions) (Ribéreau-Gayon et al. 2006). The first stage of noble rot infection, called ‘marron pourri plein’, corresponds to the penetration of the fungus into ripe berries without altering the fruit skin. The further evolution of the botrytization process seems to depend on particular weather conditions—alternations of cold humid nights and sunny dry days. The fungus induces the decomposition of the fruit skin that becomes porous. Water losses by natural evaporation then lead to intense enzymatic maceration and increase sugar concentration in berries (‘marron roti’ stage). Botrytized berries are harvested one by one by trained people at the ‘marron roti’ stage when the fungus has been killed by the increase in sugar concentration. The chemical composition between botrytized and gray molded berries differs as regards amines, conjugated polyamines, and hydroxycinnamic acids (Geny et al. 2003; Kiss et al. 2006). The noble rot also has a direct impact on aroma precursors and volatile thiols (Tominaga et al. 2006; Thibon et al. 2009), related to the characteristic exceptional range of aromas of sweet wines. At early stages of the botrytization process, a reversion of noble rot toward gray mold can be observed (G. Barbeau, Y. Brygoo, pers. com.). Moreover, both noble rot and gray mold symptoms are observed in sympatry, in the same vineyards, on the same plant, and sometimes within the same grapes. The question, however, remains whether the different symptoms are caused by genetically differentiated strains with contrasted metabolisms or by the same strains that would display different symptoms depending on environmental conditions, such as microclimatic temperature and humidity within the grape, maturity level of the infected berries, or presence of other microbial communities.

*Botrytis cinerea* (teleomorph *Botryotinia fuckeliana*) has a wide host range and can infect more than 220 eudicot plants. *Botrytis cinerea* has recently been shown to be a complex of two sibling species growing in sympatry (Fournier et al. 2005): *B. cinerea* (*sensu stricto*), *aka* ‘*B. cinerea* Group II’, is the predominant and most damageable species on grapevine, especially at vintage. The other species, initially designed *B. cinerea* Group I (Fournier et al. 2005) and recently re-named *B. pseudocinerea* (Walker et al. 2011), is found at low frequencies in French populations (Giraud et al. 1997; Walker et al. 2011). Population genetics studies have revealed the existence of regular recombination within each species (Giraud et al. 1997; Fournier et al. 2005) and have shown a significant level of genetic differentiation between *B. cinerea* Group II populations occurring on different host plants (Fournier and Giraud 2008; Karchani-Balma et al. 2008).

No attempt has been made so far to test the existence of genetic differentiation between strains causing noble rot and gray mold symptoms. This knowledge could yet have important agronomic applications. In case no genetic differentiation is found between noble rot and gray mold isolates, winegrowers should focus on the control of environmental parameters to optimize the development of noble rot. If the two symptoms were caused by two differentiated populations, noble rot could be distinguished from gray mold isolates, and specifically inoculated into targeted vineyards; this would allow the development of noble rot on more berries, increasing yield and saving the money spent to hire trained workers sorting the different kinds of infections berry by berry. It would also allow the development of new business aiming at producing noble rot inocula both for the domestic market and for export in wine-growing regions where botrytized berries are not used in the production of sweet wines. Such inoculations of ‘noble rot isolates’ could also allow producing sweet wines in a variety of regions where it could not be elaborated so far. Given the socioeconomic importance of sweet wines, this would have many applied consequences. Furthermore, various sweet wines are produced in different regions of France (e.g., Alsace and Bordelais), with debates regarding the involvement of noble rot in certain regions. The identification of genetically differentiated strains, between noble rot and gray mold or even between different regional types of noble rot, would have applied consequences, especially in terms of AOC (‘appellation d'origine contrôlée’), that is*.,* authorization of labels such as ‘noble rot’. Genetic tests could be designed and demanded for AOC labels if different types of strains are found, and further investigations could be conducted to determine the metabolic differences among noble rot regional types.

The aims of this study are therefore (i) to determine whether noble rot symptoms are caused by *B. cinerea* (*sensu stricto,* i.e., Group II) or by a cryptic distinct species, (ii) in case noble rot symptoms are caused by *B. cinerea sensu stricto*, to investigate whether genetic differentiation exists between populations of *B. cinerea* causing respectively gray mold and noble rot symptoms, and (iii) to investigate genetic differentiation between populations from different French vineyards used for sweet wine production. To achieve these goals, we sampled *Botrytis* on grapevine at fall in three French vineyards where sweet wines are produced: Alsace (Haut-Rhin, Eastern France), Anjou (Maine-et-Loire, central western France), and Bordelais (Gironde, south-western France), on berries displaying gray mold and on berries displaying noble rot symptoms in each vineyard, and we used multilocus microsatellite genotyping to investigate genetic structure.

## Material and methods

### *Botrytis* sampling

Isolates were sampled in September and October 2002 in three French regions: Alsace near the city of Colmar, Anjou near the town of Beaulieu-sur-Layon, and Bordelais near the town of Pessac (Table 1). In each region, several fields were sampled, the maximum distance between two fields never exceeding 2 km. In each field, *Botrytis* was sampled on grape (*Vitis vinifera*), both from gray mold symptoms (‘Gray Mold’ isolates) and from noble rot symptoms (‘Noble Rot’ isolates). All samples were taken from sporulating necroses on fruits using sterilized cotton buds. Samples were genetically purified by three consecutive cultures (3 days at 21°C on PDA medium) of uncontaminated growing mycelial colonies, as described in the study by Fournier and Giraud (2008). Isolates were then grown toward sporulation, and spore suspensions were stored at −80°C in 20% glycerol.

**Table 1:** Sample sizes, various indexes of genetic diversities, and linkage disequilibrium, in total and for each region × symptom combination. *N*: Number of isolates genotyped. *G*: number of observed multilocus genotypes. *G/N*: fraction of nonrepeated genotypes in the sample. *H*_e_: nonbiased genic diversity. *n*_a_: mean number of alleles per locus (standard deviation between brackets). *r*_D_: multilocus linkage disequilibrium (*P*: associated probability estimated after 500 randomizations)

Region	Symptom	*N*	*G*	*G/N*	*H*_e_	*n*_a_	*r*_D_
Anjou	Gray mold	35	34	0.97	0.51	4.4 (2.1)	0.03 (*P* = 0.03)
	Noble rot	34	33	0.97	0.48	4.6 (2)	−0.02 (*P* = 0.66)
Bordelais	Gray mold	38	36	0.95	0.46	4 (2)	0.02 (*P* = 0.08)
	Noble rot	18	17	0.94	0.37	2.7 (2)	0.02 (*P* = 0.22)
Alsace	Gray mold	18	18	1	0.57	4.5 (2.1)	−0.01 (*P* = 0.65)
	Noble rot	21	21	1	0.56	4.1 (1.9)	0.01 (*P* = 0.24)
Total		164	153	0.93	0.51	6.4 (3.1)	0.01 (*P* = 0.46)

### DNA extraction and microsatellite amplification

For each isolate, genomic DNA was extracted using the Chelex (Bio-Rad laboratories, Hercules, CA, USA) protocol described in the study by Bucheli et al. (2001). Eight microsatellite markers (Fournier et al. 2002) were amplified, with forward primers labeled with a fluorescent probe. PCR amplifications were performed as in Fournier et al. (Fournier et al. 2002) and gel visualization as in Giraud (2004). The *Bc6* locus carries a diagnostic allele for *B. pseudocinerea* (Fournier and Giraud 2008; Walker et al. 2011).

### Genic and genotypic diversities, and linkage disequilibrium

The software fstat version 2.9.3 (Goudet 1995) was used to estimate the unbiased genic diversity, *H*_e_ (Nei 1987), and the mean number of alleles per locus, *n*_a_*,* over all loci in the total sample, in each of the six regionX symptom combination and in each cluster inferred using assignment methods. fstat was also used to calculate pairwise Weir and Cockerham's *F*_ST_ (Weir and Cockerham 1984) between the clusters inferred using assignment methods and to evaluate their significance by random permutations and if necessary by progressive Bonferroni correction as described by Rice (1989). The number of different multilocus genotypes, *G*, the haplotypic diversity, and the index of association, *r*_D_, were calculated using Multilocus 1.3b (Agapow and Burt 2001). The *r*_D_ index measures the multilocus linkage disequilibrium (Brown et al. 1980; Maynard-Smith et al. 1993; Haubold et al. 1998) and indicates whether two different individuals sharing the same allele at one locus are more likely to share the same allele at another locus, based on the variance of the number of loci with respect to which two individuals differ. *r*_D_ is insensitive to the number of scored loci and varies from 0 (complete panmixia) to 1 (complete linkage disequilibrium). The null hypothesis of panmixia (*r*_D_ = 0) was tested using the reshuffling procedure implemented in the software (500 randomizations).

### Analyses of population subdivision

We used Structure version 2.3.1 (Pritchard et al. 2000) to examine population subdivision. The model implemented allowed admixture and correlation in allele frequencies (Falush et al. 2003). Burn-in length was set at 50 000 iterations followed by a run phase of 500 000 iterations. Analyses were conducted both using uniform priors and using the region of origin of isolates or type of symptoms as prior information to assist clustering (LocPrior model; Hubisz et al. 2009). We set the number of populations (*K*) from 1 to 8, but not higher, as preliminary analyses revealed that setting *K* values above 8 led to clusters with highly admixed ancestry, which is typical of too high a cluster number. For all analyses, we performed 30 independent runs for each value of *K*. Outputs were processed using clumpp version 1.1.2 (Jakobsson and Rosenberg 2007) to identify distinct modes in the replicated runs of each *K* (‘modes’ refer to distinct clustering solutions). We computed the rate of change in the log probability of data between successive *K* values (*ΔK* statistic; Evanno et al. 2005).

We also used the discriminant analysis of principal components (DAPC, Jombart et al. 2010) as an alternative method to infer population subdivision. This multivariate method consists of a discriminant analysis on data transformed after a principal component analysis. We used the *adegenet* package (1.3-1) of the R 2.13.1 software (R Foundation for Statistical Computing, Vienna, Austria, http://www.R-project.org) to perform DAPC analyses. The K-means procedure implemented in the function *find.cluster* allows determining the optimal number of clusters, *C*. We let *C* vary between 1 and 50 (with 5000 iterations for each value of *C*) and used the Bayesian information criterion (BIC) to determine the optimal value of *C* (corresponding to the value of *C* for which the BIC is minimal or at which the rate of change in the BIC abruptly changes).

## Results

In total, 164 individual genotypes were analyzed, with sample sizes ranging between 18 and 38 in the different region × symptom combinations (Table 1). The data set presented 4.2% of missing data, corresponding to 55 individuals lacking information at one locus at most. On the basis of the diagnostic allele at the *Bc6* locus, all individuals were identified as *B. cinerea sensu stricto* (Group II). Both in the full data set and in the region × symptom subsamples, most multilocus genotypes (MLGs) obtained with the eight microsatellites were each detected in a single individual, resulting in a *G/N* ratio of 0.93 in total and never lower than 0.94 within subsamples (*G*: number of different MLG, *N*: sample size). Haplotypic diversity was 0.998 overall and ranged from 0.993 to 1 in subsamples. Nei's genic diversity *H*_e_ was 0.51 in the full data set and varied within subsamples between 0.37 (Bordelais) and 0.57 (Alsace). Multilocus linkage disequilibrium was low in all subsamples (*r*_D_ ranging from 0 to 0.03) and never significantly different from 0 (except for the Anjou Gray subsample), indicating no detectable departure from panmixia.

**Figure 1:**
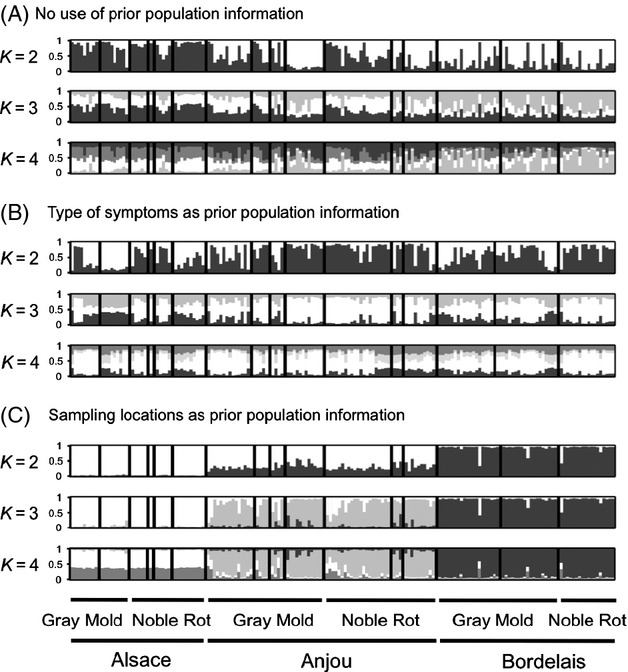
Barplots of the Structure analysis without any prior information (A), with the symptom as prior information (B) or with the geographic origin as prior information (C).

Structure results are presented in Fig. 1. Only the membership coefficients for runs with 2 ≤ *K* ≤ 4 are shown, because higher values of *K* revealed no new clusters and only introduced some heterogeneity into membership coefficients. Analyses that made no use of prior population information or that used the type of symptoms (« Gray Mold » versus « Noble Rot ») to assist clustering revealed no clear pattern of clustering of genotypes (Figs 1A and B). The modal value of the *ΔK* statistic was observed at *K* = 2 for these models of population structure. However, the barplots showed pervasive admixture, providing little evidence for the existence of distinct clusters. In the case of the analysis using the type of symptom as prior information, the 1st cluster encompassed 65% of gray mold isolates and the 2nd cluster encompassed 57% of gray mold isolates. The assignment probability of individuals in the two inferred clusters depending on the type of symptom did not depart from random (χ^2^ = 0.59, *P* = 0.44, df = 1).

In contrast, analyses that used sampling locations as prior population information revealed a clear geographic pattern of clustering (Fig. 1C). Genotypes from Alsace, Anjou, and Bordelais had high membership values in distinct clusters (average ± standard deviation (%): 94.5 ± 3.8, 73.1 ± 22.3, 93.3 ± 10.5, respectively). The modal value of the *ΔK* statistic was observed at *K* = 3. The interclusters *F*_ST_ ranged from 5% (between Anjou and Bordelais clusters) to 11–12% (between Alsace and the two other clusters). Hence, the partition of genetic diversity of *B. cinerea* populations is better explained by geography than by the type of symptom.

**Figure 2:**
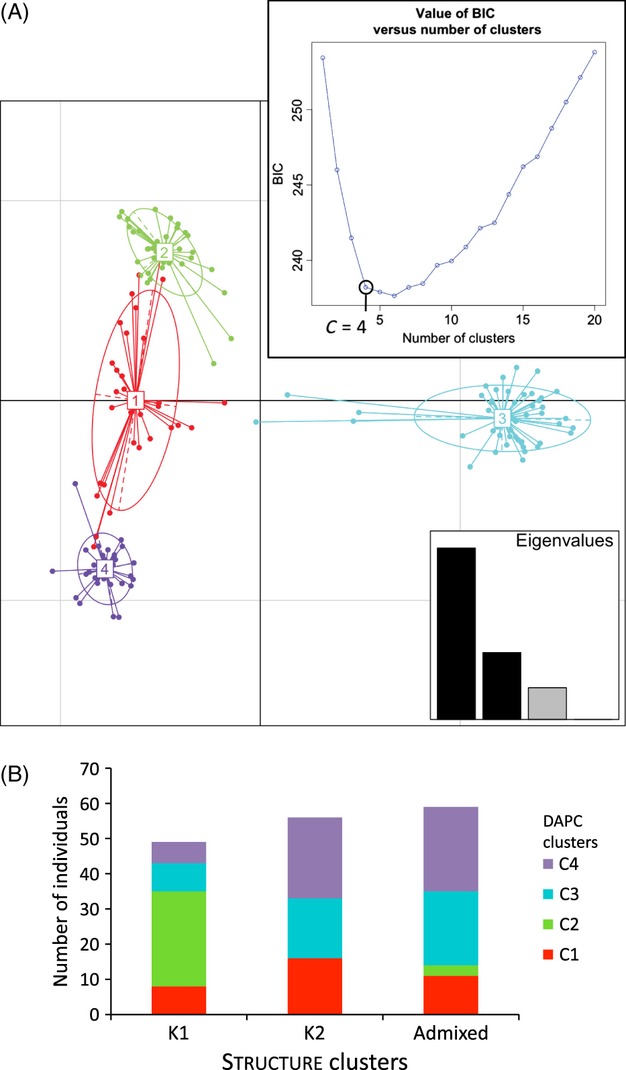
Scatter plot of the DAPC analysis without any prior information (A), and comparison of individual assignment between DAPC and Structure analyses without prior information (B). Individuals are considered assigned to a cluster if their posterior probability in that cluster is at least 0.75.

**Figure 3:**
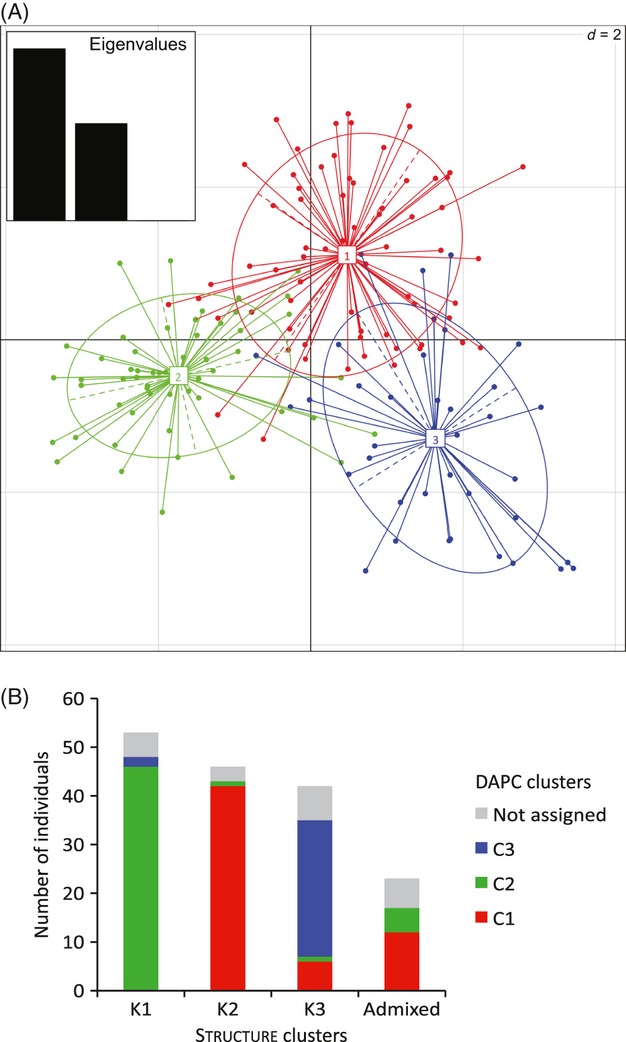
Scatter plot of the DAPC analysis with the geographic origin of isolates as prior information (A), and comparison of individual assignment between DAPC and Structure analyses with geography as prior information (B). Individuals are considered assigned to a cluster if their posterior probability in that cluster is at least 0.75.

To confirm the population subdivision inferred using Structure, we performed a DAPC analysis, first without any *a priori* information on the origin of individuals or their type of symptoms (Fig. 2). The significant number of DAPC groups given by the K-means procedure was *C* = 4 (Fig. 2A). The pairwise *F*_ST_ between these four DAPC groups ranged from 0.15 to 0.20. Individual assignments provided by DAPC differed from those by Structure: one of the Structure clusters was split into three DAPC groups, while the fourth DAPC group was composed of 55% of the individuals assigned to the second Structure cluster (Fig. 2B). The lack of congruence between the two approaches strengthens the conclusion that there is no strong pattern of population subdivision that would be detectable without using *a priori* knowledge to assist clustering. We then performed DAPC with three groups defined *a priori* on the basis of the geographic origin of individuals. The posterior assignments confirmed the relevance of the use of the geographic prior information: 80% of individuals sampled in Anjou were assigned to a first DAPC group, 84% of individuals sampled in Bordelais were assigned to the second DAPC group, and 72% of individuals sampled in Alsace were assigned to the third DAPC group (Fig. 3A). These DAPC assignments fitted well with the assignments obtained with the Structure analyses using geographic information to assist clustering (Fig. 3B).

**Figure 4:**
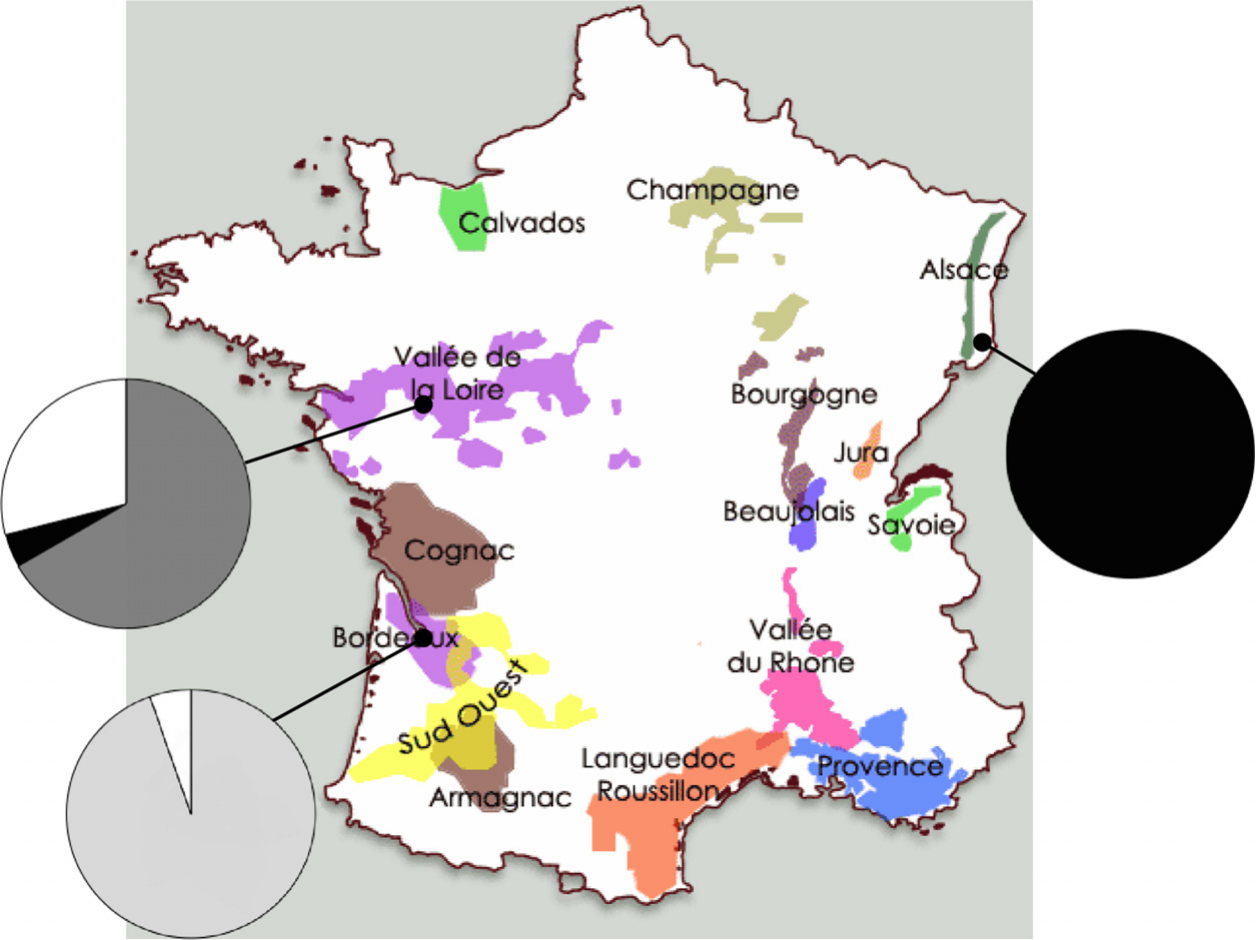
Proportions of individuals assigned to the three clusters inferred with Structure using geographic prior information, in each of the three sampled regions. Light gray: cluster K1, dark gray: cluster K2, black: cluster K3, white: admixed individuals. The different French vineyards are indicated.

The assignment methods using the geographic origin of isolates as prior information thus partitioned *B. cinerea* populations into three clusters strongly associated with the geographic origin of isolates (Table 2, Fig. 4). The first cluster, composed of 53 isolates exclusively coming from Bordelais, encompassed the highest proportion of repeated genotypes (*G/N* = 0.81) and was the less diverse (genic diversity *H*_e_ = 0.414, mean number of alleles per locus *n*_a_ = 3.5). The second cluster counted 46 isolates, all from the Anjou region, in which all MLGs but one were represented only once (*G/N* = 0.98). This cluster had a genic diversity *H*_e_ of 0.448 and a mean number of alleles per locus *n*_a_ of 3.6. The 42 isolates assigned to the third cluster came predominantly from Alsace (39/42) and were all unique MLGs. This 3rd cluster was the most diverse one (*H*_e_ = 0.581, *n*_a_ = 5.7). All three clusters were characterized by low values of multilocus linkage disequilibrium (*r*_D_ ranging from 0.002 to 0.008). The Alsace cluster was also the most strongly differentiated from the two others (pairwise *F*_ST_*:* Alsace-Bordelais = 0.12, Alsace-Anjou = 0.11, Bordelais-Anjou = 0.05).

**Table 2:** Sizes of the clusters inferred using Structure, various indexes of genetic diversities and linkage disequilibrium within each cluster. Admixed individuals have posterior probabilities not higher than 0.75 in any of the clusters. The main geographic origin in the cluster gives the percentage of isolates assigned to this cluster (with a posterior probability not lower than 0.75) coming from a particular region. *N*: Number of isolates genotyped. *G*: number of observed multilocus genotypes. *G/N*: fraction of nonrepeated genotypes in the sample. *H*_e_: nonbiased genic diversity. *n*_a_: mean number of alleles per locus (standard deviation between brackets). *r*_D_: multilocus linkage disequilibrium (*P*: associated probability estimated after 500 randomizations)

Cluster	Main geographic origin in the cluster	*N*	*G*	*G/N*	*H*_e_	*n*_a_	*r*_D_
Cluster 1	Bordelais (100%)	53	43	0.81	0.414	3.5 (1.9)	0.002 (*P* = 0.37)
Cluster 2	Anjou (100%)	46	45	0.98	0.448	3.6 (1.8)	0.008 (*P* = 0.24)
Cluster 3	Alsace (93%)	42	42	1	0.581	5.7 (2.6)	0.002 (*P* = 0.41)
Admixed		23					

## Discussion

Our study shows that noble rot symptoms on grapevine are caused by *B. cinerea* (*sensu stricto*) and not by *B. pseudocinerea* or by another cryptic species. The two clusters inferred using the Structure program without making use of sample group information to assist clustering were explained neither by the type of symptom nor by the geographic origin of isolates. This indicates weak population structure. Such limitations of the Structure method have recently been pinpointed: as this method considers all partitions as equally likely, a considerable amount of statistical evidence is needed to provide strong support for any particular partition, which is impossible to obtain when the data contain relatively little information (Hubisz et al. 2009). This justifies the use of group information to assist the clustering algorithm. In the present case, prior knowledge on symptom caused by the sampled isolates did not reveal any structure. In contrast, making use of the geographic origin of individuals considerably improved the Structure clustering and led to a subdivision into three clusters clearly explained by geography. The confidence in this pattern of geographic subdivision was reinforced by the congruent results obtained using a DAPC analysis making use of sampling locations as prior information.

The cluster grouping isolates from Alsace was the most diverse one, showing the highest genetic diversity and the highest number of alleles per locus. The ‘Alsace’ cluster was also the most strongly differentiated from the two other ones. The ‘Alsace’ vineyard is geographically disconnected from the other French wine-producing regions, especially due to the Vosges Mountains, while being connected to West German vineyards. Anjou and Bordelais vineyards are in contrast more or less interconnected. The populations in Anjou and Bordelais experience different climatic conditions than those in Alsace. However, these differences in abiotic selective pressures are unlikely to be strong enough to lead to immigrant inviability or to the evolution of extrinsic postzygotic barriers to gene flow, which would be necessary to generate genetic differentiation at neutral loci (Giraud et al. 2010). The observed population subdivision may rather be due to the lesser connectivity between Alsace and the two other regions. Also, as already outlined, significant population differentiation among host plants exists in *B. cinerea* (Fournier and Giraud 2008), likely due to specialization to the host, but with significant gene flow. We therefore cannot exclude that the different geographic regions host populations of *B. cinerea* specialized on different plants, which would mix on different proportions into the population sampled on grape, therefore leading to significant differences in allelic frequencies in the analyzed samples due to host specialization instead of geographic isolation.

The overall level of genetic differentiation appears to be quite low, with pairwise *F*_ST_ ranging from 5 to 12%. Such a pattern of weak genetic structure in *B. cinerea* has been previously reported (Fournier and Giraud 2008) and indicates a history of recent continental spread, large scale contemporary gene flow and/or large effective population sizes. Our study also confirmed previous results showing that there was no linkage disequilibrium in *B. cinerea* populations (Giraud et al. 1997; Fournier and Giraud 2008; Karchani-Balma et al. 2008). Hence, although sexual reproduction has never been observed so far *in natura*, *B. cinerea* populations experience apparently regular events of recombination.

To conclude, gray mold and noble rot symptoms on grapevine are both caused by the same species, *B. cinerea* (*sensu stricto*), and there is no genetic differentiation, hence no barrier to gene flow, between isolates causing the different symptoms. This raises the question of the proximal mechanisms inducing the ‘noble’ pathway in *B. cinerea*. This fungus is able to attack a wide range of host species, if the conditions are met (Elad and Stewart 2004; Choquer et al. 2007; Williamson et al. 2007). Besides encompassing a large arsenal of genes involved in necrotrophic processes (such as cell wall degradation or oxalic acid production), the genome of *B. cinerea* is enriched in secondary metabolism gene clusters and also harbors 5% (879/16400) genes encoding secreted proteins (Amselem et al. 2011). Recently, it has been shown that *B. cinerea* has a stimulating effect in the production of volatile thiols—compounds naturally produced by the grape berry that contribute strongly to the varietal aromas (Thibon et al. 2009, 2011). More precisely, the 100- to 1000-fold enrichment of one particular cysteine S-conjugate precursors in botrytized berries was due to the production of metabolites by the fungus that were likely unstable in time and temperature-sensitive (Thibon et al. 2011). Therefore, this metabolic pathway involved in the botrytization process is likely to be modulated by microclimatic conditions. This hypothesis is in accordance with the field observation that the switch between gray mold and noble rot tightly depends on microclimatic conditions; a better description and understanding of the humidity and temperature conditions required for noble rot development would then be useful. We cannot rule out the existence of genetic differentiation at one (or a few) gene(s), possibly involved in the activation and/or regulation of this kind of metabolic pathways driving aromatic compounds biosynthesis. Further functional and transcriptomic studies are needed to investigate the existence of such genes and seek for a link between genotypes and phenotypes at these loci. Functional and transcriptomic studies could also help reveal which metabolic pathways are involved in the switch between the types of symptoms. Our study therefore strongly prompts for such studies: identifying a particular metabolic pathway and its mode of regulation could open the way for optimizing the production of sweet wines. Being able to foster noble rot symptoms and prevent the switch to gray mold would have important applied consequences and now appears the best route for optimizing the production of botrytized berries. Our study revealed the existence of genetic differentiation between regions, which may allow designing PCR-based tests on wines for checking the geographic origins in the context of AOC labels.

## Data archiving statement

Data deposited in the Dryad repository: doi:10.5061/dryad.6mv07.

## References

[b1] Agapow P-M, Burt A (2001). Indices of multilocus linkage disequilibrium. Molecular Ecology Notes.

[b2] Amselem J, Cuomo CA, Viaud JAL, van Kan M, Benito EP, Couloux A, Coutinho PM (2011). Genomic analysis of the necrotrophic fungal pathogens *Sclerotinia sclerotiorum* and *Botrytis cinerea*. PLOS Genetics.

[b3] Brown AHD, Feldman MW, Nevo E (1980). Multilocus structure of natural populations of *Hordeum spontaneum*. Genetics.

[b4] Bucheli E, Gautschi B, Shykoff JA (2001). Differences in population structure of the anther smut fungus Microbotryum violaceum on two closely related host species, *Silene latifolia* and *S. dioica*. Molecular Ecology.

[b5] Choquer M, Fournier E, Kunz C, Levis C, Pradier J-M, Simon A, Viaud M (2007). *Botrytis cinerea* virulence factors: new insights into a necrotrophic and polyphageous pathogen. FEMS Microbioly Letters.

[b6] Dettman JR, Jacobson DJ, Taylor JW (2003a). A multilocus genealogical approach to phylogenetic species recognition in the model eukaryote *Neurospora*. Evolution.

[b7] Dettman JR, Jacobson DJ, Turner E, Pringle A, Taylor JW (2003b). Reproductive isolation and phylogenetic divergence in Neurospora: comparing methods of species recognition in a model eukaryote. Evolution.

[b8] Elad Y, Stewart A, Elad Y, Williamson B, Tudzynski P, Delen N (2004). Microbial control of *Botrytis* spp. Botrytis: Biology, Pathology and Control.

[b9] Evanno G, Regnaut S, Goudet J (2005). Detecting the number of clusters of individuals using the software structure: a simulation study. Molecular Ecology.

[b10] Falush D, Stephens M, Pritchard J (2003). Inference of population structure using multilocus genotype data: linked loci and correlated allele frequencies. Genetics.

[b11] Fitt B, Huang Y-J, West F, van den Bosch JS (2006). Coexistence of related pathogen species on arable crops in space and time. Annual Review of Phytopathology.

[b12] Fournier E, Giraud T (2008). Sympatric genetic differentiation of a generalist pathogenic fungus, *Botrytis cinerea*, on two different host plants, grapevine and bramble. Journal of Evolutionary Biology.

[b13] Fournier E, Giraud T, Loiseau A, Vautrin D, Estoup A, Solignac M, Cornuet JM (2002). Characterization of nine polymorphic microsatellite loci in the phytopathogenic fungus *Botrytis cinerea* (Ascomycota). Molecular Ecology Notes.

[b14] Fournier E, Lévis C, Fortini D, Giraud T, Leroux P, Brygoo Y (2003). Characterization of Bc-*hch*, the *Botrytis cinerea* homolog of the *Neurospora crassa het-c* vegetative incompatibility locus, and its use as a population marker. Mycologia.

[b15] Fournier E, Giraud T, Brygoo Y (2005). Partition of the *Botrytis cinerea* complex in France using multiple gene genealogies. Mycologia.

[b16] Gentetaki E, Lynn DH (2010). Evidence for cryptic speciation in *Carchesium polypinum* Linnaeus, 1758 (Ciliophora: Petrichia) inferred from mitochondrial, nuclear, and morphological markers. Journal of Eukaryotic Microbiology.

[b17] Geny L, Darrieumerlou A, Donèche B (2003). Conjugated polyamines and hydroxycinnamic acids in grape berries during Botrytis cinerea disease development: differences between ‘noble rot’ and ‘grey mould’. Australian journal of grape and wine research.

[b18] Giraud T (2004). Patterns of within population dispersion and mating of the fungus *Microbotryum violaceum* parasitising the plant *Silene latifolia*. Heredity.

[b19] Giraud T, Fortini D, Levis C, Leroux P, Brygoo Y (1997). RFLP markers show genetic recombination in *Botryotinita fuckeliana**Botrytis cinerea*) and transposable elements reveal two sympatric species. Molecular Biology and Evolution.

[b20] Giraud T, Refrégier G, Hood DM, de Vienne MA, Le Gac ME (2008). Speciation in fungi. Fungal Genetics and Biology.

[b21] Giraud T, Gladieux P, Gavrilets S (2010). Linking emergence of fungal plant diseases and ecological speciation. Trends in Ecology & Evolution.

[b22] Gladieux P, Caffier V, Devaux M, Le Cam B (2010). Host-specific differentiation among populations of *Venturia inaequalis* causing scab on apple, pyracantha and loquat. Fungal Genetics and Biology.

[b23] Gladieux P, Byrnes E, Fisher MC, Aguileta G, Heitman J, Giraud T, Tibayrenc M (2011a). Epidemiology and evolution of fungal pathogens, in plants and animals. Genetics and Evolution of Infectious Diseases.

[b24] Gladieux P, Vercken E, Fontaine MC, Hood ME, Jonot O, Couloux A, Giraud T (2011b). Maintenance of fungal pathogen species that are specialized to different hosts: allopatric divergence and introgression through secondary contact. Molecular Biology and Evolution.

[b25] Goudet J (1995). FSTAT (Version 1.2): a computer program to calculate F-statistics. Heredity.

[b26] Haubold B, Travisiano PB, Hudson RR (1998). Detecting linkage disequilibrium in bacterial populations. Genetics.

[b27] Heimeier D, Lavery S, Sewell MA (2010). Molecular species identification of *Astrotoma agassizii* from planktonic embryos: further evidence for a cryptic species complex. Journal of Heredity.

[b28] Hubisz MJ, Falush D, Stephens M, Pritchard J (2009). Inferring weak population structure with the assistance of sample group information. Molecular Ecology Resources.

[b29] Jakobsson M, Rosenberg NA (2007). CLUMPP: a cluster machine and permutation program for dealing with label switching and multimodality in analysis of population structure. Bioinformatics.

[b30] Jesse R, Schubart CD, Klaus S (2010). Identification of a cryptic lineage within *Potamon fluviatile* (Crustacea: Brachyura: Potamidae). Invertebrate Systematics.

[b31] Johnson EM (2009). Rare and emerging *Candida* species. Current Fungal Infection Reports.

[b32] Jombart T, Devillard S, Balloux F (2010). Discriminant analysis of principal components: a new method for the analysis of genetically structured populations. BMC Genetics.

[b33] Karchani-Balma S, Gautier A, Raies A, Fournier E (2008). Geography, plants, and growing systems shape the genetic structure of Tunisian *Botrytis cinerea* populations. Phytopathology.

[b34] Kiss J, Korbasz M, Sass-Kiss A (2006). Study of amine composition of botrytized grape berries. Journal of agricultural and food chemistry.

[b35] Koufopanou V, Burt A, Taylor JW (1997). Concordance of gene genealogies reveals reproductive isolation in the pathogenic fungus *Coccidioides immitis*. PNAS.

[b36] Kreier HP, Feldberg K, Mahr F, Bombosch A, Schmidt AR, Zhu RL, von Konrat M (2010). Phylogeny of the leafy liverwort *Ptilidium*: cryptic speciation and shared haplotypes between the Northern and the Southern hemispheres. Molecular Phylogenetics and Evolution.

[b37] Kuehne HA, Murphy HA, Francis CA, Sniegowski PD (2007). Allopatric divergence, secondary contact and genetic isolation in wild yeast. Current Biology.

[b38] Le Cam B, Parisi L, Arene L (2002). Evidence of two formae speciales in *Venturia inaequalis*, responsible for apple and pyracantha scab. Phytopathology.

[b39] Le Gac M, Hood ME, Fournier E, Giraud T (2007a). Phylogenetic evidence of host-specific cryptic species in the anther smut fungus. Evolution.

[b40] Le Gac M, Hood ME, Giraud T (2007b). Evolution of reproductive isolation within a parasitic fungal complex. Evolution.

[b41] Liu J, Moller M, Gao LM, Zhang D-Q, Li D-Z (2011). DNA barcoding for the discrimination of Eurasian yews (Taxus L., Taxaceae) and the discovery of cryptic species. Molecular Ecology Resources.

[b42] Maynard-Smith J, Smith NH, O'Rourke M, Spratt BG (1993). How clonal are bacteria ?. Proceedings of the National Academy of Sciences of the United States of America.

[b43] Nei M (1987). Molecular Evolutionary Genetics.

[b44] Nygren A, Eklof J, Pleijel F (2010). Cryptic species of Notophyllum (Polychaeta: Phyllodocidae) in Scandinavian waters. Organisms diversity and evolution.

[b45] O'Donnell K, Ward T, Geiser DM, Kistler HC, Aoki T (2004). Genealogical concordance between the mating type locus and seven other nuclear genes supports formal recognition of nine phylogenetically distinct species within the *Fusarium graminearum* clade. Fungal Genetics and Biology.

[b46] Peman J, Canton E, Quindos G, Eraso E, Alcoba J, Guinea J, Merino P (2012). Epidemiology, species distribution and *in vitro* antifungal susceptibility of fungaemia in a Spanish multicentre prospective survey. Journal of Antimicrobial Chemotherapy.

[b47] Piggott MP, Chao NL, Beheregaray LB (2011). Three fishes in one: cryptic species in an Amazonian floodplain forest specialist. Biological Journal of the Linnean Society.

[b48] Pritchard JK, Stephens P, Donnelly P (2000). Inference of population structure using multilocus genotype data. Genetics.

[b49] Ribéreau-Gayon P, Dubourdieu D, Donèche B, Lonvaud A (2006). Handbook of Enology. The Microbiology of Wine and Vinifications.

[b50] Rice WR (1989). Analyzing tables of statistical tests. Evolution.

[b51] Schneider S, Rehner SA, Widmer F, Enkerli J (2011). A PCR-based tool for cultivation-independent detection and quantification of Metarhizium clade 1. Journal of Invertebrate Pathology.

[b52] Taylor JW, Jacobson DJ, Kroken S, Kasuga T, Geiser DM, Hibbett DS, Fisher MC (2000). Phylogenetic species recognition and species concepts in fungi. Fungal Genetics and Biology.

[b53] Thibon C, Dubourdieu D, Darriet P, Tominaga T (2009). Impact of noble rot on the aroma precursor of 3-sulfanylhexanol content in Vitis vinifera L. cv Sauvignon blanc and Semillon grape juice. Food Chemistry.

[b54] Thibon C, Cluzet S, Mérillon J-M, Darriet P, Dubourdieu D (2011). 3-Sulfanylhexanol precursor biogenesis in grapevine cells: the stimulating effect of *Botrytis cinerea*. Journal of Agricultural and Food Chemistry.

[b55] Tominaga T, Niclass Y, Frérot E, Dubourdieu D (2006). Stereoisomeric distribution of 3-mercaptohexan-1-ol and 3-mercaptohexyl acetate in dry and sweet white wines made from *Vitis vinifera* (Var. Sauvignon Blanc and Semillon). Journal of agricultural and food chemistry.

[b56] Walker A-S, Gautier A, Confais J, Martinho D, Viaud M, Dupont P, Le Pêcheur J (2011). *Botrytis pseudocinerea*, a new cryptic species causing grey mould in French vineyards in sympatry with *Botrytis cinerea*. Phytopathology.

[b57] Weir BS, Cockerham CC (1984). Estimating F-statistics for the analysis of population structure. Evolution.

[b58] Williamson B, Tudzynski B, Tudzynski P, Van Kan J (2007). *Botrytis cinerea*: the cause of grey mould disease. Molecular Plant Pathology.

